# Climate Impact of Laryngeal Masks: Climate and Other Environmental Impacts of Reusable and Single‐Use Laryngeal Masks in Sweden

**DOI:** 10.1111/aas.70144

**Published:** 2025-11-04

**Authors:** Adrien Talbot, Gang Liang, Andrius Plepys, Peter Bentzer

**Affiliations:** ^1^ Department of Anaesthesiology and Intensive Care Skåne University Hospital Helsingborg Sweden; ^2^ Department of Clinical Sciences Lund University Lund Sweden; ^3^ Department of Community Development Environmental Division, Trelleborg Municipality Trelleborg Sweden; ^4^ International Institute for Industrial Environmental Economics Lund University Lund Sweden

**Keywords:** anaesthesia, aura40, aurastraight, climate change, greenhouse gas emissions, healthcare, igel, laryngeal mask airway, life cycle assessment, reusable

## Abstract

**Background:**

The use of single‐use laryngeal masks has increased in recent decades; yet, their climate and environmental impacts remain poorly understood. This study aimed to compare the climate impacts of reusable and single‐use laryngeal masks.

**Methods:**

We conducted a life cycle assessment that compared the reusable Ambu Aura40 with the single‐use Ambu AuraStraight and Intersurgical Igel+ laryngeal masks. Data were obtained from the manufacturers, the ecoinvent database v3.10 and from Helsingborg Hospital, Sweden. Climate impacts were assessed using the ReCiPe2016 method, assuming 40 reuses of the Aura40. The results were expressed in carbon dioxide equivalents over a 100‐year period (CO_2_e) and presented as medians with 95% reference intervals for one use.

**Results:**

The climate impact of the Aura40 was 141 gCO_2_e (129–156), versus 597 gCO_2_e (533–686) and 1000 gCO_2_e (848–1210) for the AuraStraight and Igel+, respectively. Compared with the Aura40, the AuraStraight had a 323% greater climate impact (∆456 gCO_2_eq [390–535]), and the Igel+ had a 607% higher climate impact (∆856 gCO_2_eq [709–1070]). The Igel+ had a 68% greater climate impact (∆404 gCO_2_eq [224–608]) than the AuraStraight. Sensitivity analyses indicated that the climate impact of the Aura40 exceeded that of the AuraStraight and was similar to that of the Igel+ when a high‐climate‐impact electricity mix was used.

**Conclusions:**

The Aura40 reusable laryngeal mask constitutes an opportunity to reduce the climate impact of anaesthesia in a setting with a low‐climate‐impact electricity mix. Among single‐use options, the AuraStraight had a lower climate impact than the Igel+.

**Editorial Comment:**

Single‐use or reusable anesthesia equipment, like laryngeal masks have different costs associated with their use, and leave different environmental ‘footprints’. This analysis presents models for these, to estimate the environmental impact and costs of these. This type of analysis is important to try to clarify the use ‘case’ for different alternatives for choices for materiel used in clinical areas like anesthesia.

## 1 Introduction

The laryngeal mask (LM) was introduced into clinical practice in the late 1980s to facilitate airway management in anaesthetised patients. The first LMs were reusable devices, but in the late 1990s, single‐use LMs became available and are now used widely [1].

Due to the rising number of surgeries worldwide, the global use of LMs continues to increase [2]. The rationale for the shift to single‐use devices has been driven by various factors, including perceived improvements in infection risks, lower costs and ease of use [3, 4, 5].

There is a paucity of data on the environmental effects of the transition from reusable to single‐use LMs [6]. In 2012, a life cycle assessment (LCA) study that was conducted in a North American setting compared the reusable LMA Classic with the single‐use LMA Unique and found that the reusable device had a smaller environmental footprint than the single‐use mask [7]. However, an LCA is highly context‐dependent, and input data that are relevant to North America may not be generalisable to other settings, such as Europe. Moreover, the software packages and databases that are used in LCAs have evolved significantly since that study, as have models of LMs [8]. Collectively, these factors highlight the need to assess the climate and environmental impacts of contemporary LMs in a European context.

Based on the above, the main objective of this study was to estimate and compare the climate impact of reusable and single‐use LMs in a Swedish and European setting. The secondary objective was to examine other relevant environmental impacts of LMs. For this purpose, we modelled the life cycle of different laryngeal masks using a combination of data collected locally and a generic database describing environmental impacts of identified materials and processes.

## 2 Materials and Methods

### 2.1 Objective and Scope

The main objective was to compare the life cycle climate impact of the reusable LM, Ambu Aura40 (Aura40), with our current first‐hand and second‐hand choices of laryngeal masks, the single‐use Ambu AuraStraight (AuraStraight) and the Intersurgical Igel+ (Igel+). The secondary objective was to compare the other environmental impacts. The size of the masks in this study was 5 for the AuraStraight and Aura40 and 4 for the Igel+, which are the recommended sizes for an average adult, weighing 75 kg.

A functional unit was defined as one use of a LM. For the reusable LM Aura40, 1 of 40 uses was considered, based on the manufacturer's recommendations.

### 2.2 Life Cycle Assessment

This analysis was based on an LCA, a quantitative modelling method for comparing the environmental impacts of goods, services or systems over their entire life cycle, including raw material extraction, manufacturing, transport, use and disposal. Our modelling was guided by the ISO 14044 standard and performed using the SimaPro 10.1 software package. Environmental emissions from materials, manufacturing processes of the products and waste management were drawn from the ecoinvent v3.10 LCA inventory database.

### 2.3 System Boundaries

**FIGURE 1:**
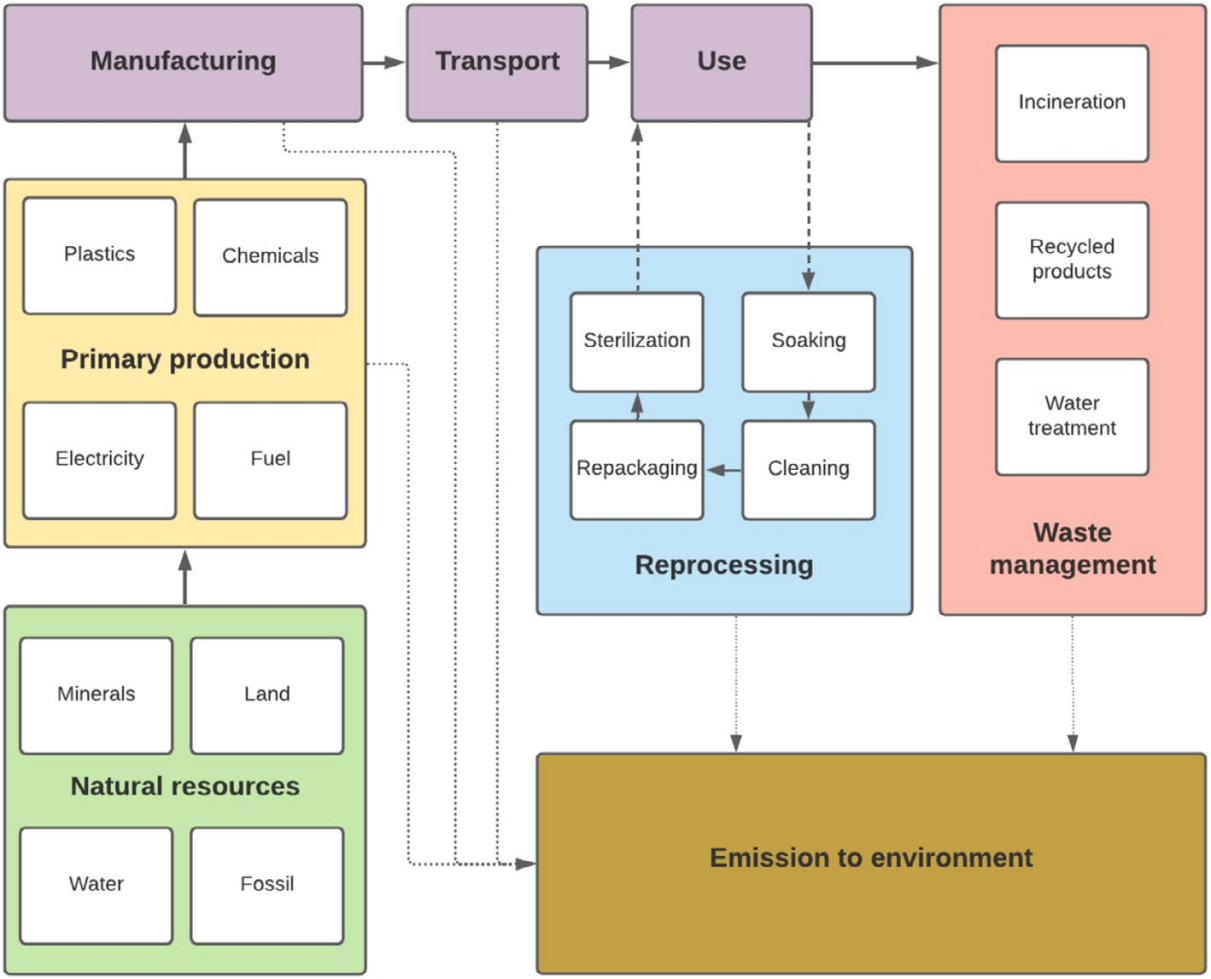
System boundaries of the life cycle analysis. The environmental impact of manufacturing capital goods, such as washing machines and sterilisers, lies outside of the system boundaries.

The system boundaries of our model are shown in Figure 1. The analysis included the production of materials; the manufacturing process; the packaging of the LMs; their transport from the manufacturing plant to Helsingborg Hospital, Sweden; the use phase (including reprocessing for reusable LMs) and waste management. Existing infrastructure was regarded as capital goods and was not included in the analysis [9, 10]. Thus, the environmental effects of transport by lorry and sterilisation were included in the analysis, but those of the manufacture of the lorry and autoclave lay outside of the system boundaries. Due to difficulties in obtaining data from the manufacturers, we did not include the plasticisers, colorants and oils that were used in the production of LMs or the industrial sterilisation of the single‐use LMs.

### 2.4 Life Cycle Inventory

Information on material composition, production, transport, energy use and disposal was obtained from the manufacturers or was based on local practices at Helsingborg Hospital, Sweden [11]. Cleaning and sterilisation processes (reprocessing) were modelled based on the manufacturers' instructions and local practices at a Swedish hospital that used these devices. Components were weighed on a scale with a precision of ±10 mg (KERN & SOHN GmbH). Transportation distances were calculated using Google Maps and sea‐distance.org. If a material was not available in ecoinvent we chose the material with the closest composition and geographic origin. The environmental emissions of chosen materials and processes in ecoinvent are based on average data and may not reflect the emissions of the actual factories.

### 2.5 Reprocessing

After each use, the Aura40 was soaked in water with detergent for 30 min and then cleaned at 90°C in a washer‐disinfector (Model 46‐4‐203, Getinge AB, Sweden). The maximum load of the washer‐disinfector was 10 LMs, and it was assumed to operate with a load of seven LMs per cycle in the primary analysis.

After the cleaning step, each LM was packaged individually in a steam‐autoclave‐proof pouch, which was then sealed using a sealing machine, consuming 0.001 kWh per seal. The entire pouch was then sterilised in a steam autoclave (HS 6617‐ER2, Getinge AB, Sweden). Although the autoclaves are not run solely with LMs, care is taken to ensure high utilisation. Each basket in the autoclave could be filled with six LMs, and a full cycle typically contains at least 10 baskets of 12, which was assumed to constitute an 83% autoclave load in our primary model.

Electricity was modelled using the most recent Swedish electricity mix in the ecoinvent database for 2020 (45% hydroelectric, 29% nuclear, 17% wind and 8% biomass/waste) [12].

### 2.6 End of Life

End‐of‐life stage was modelled according to hospital protocols. All LMs were assumed to be sent away for municipal incineration. Eighty percent of packaging materials were assumed to be recycled—as plastic or paper—and the remainder was incinerated. Wastewater and liquid effluents from cleaning processes were modelled as municipal wastewater.

### 2.7 Life Cycle Impact Assessment

We applied the ReCiPe 2016 impact assessment method, which characterises the impacts of a product on 18 environmental outcomes (Hierarchic, v1.1 global) [8, 13]. The impact assessment method sums up the physical outputs of the life cycle and allocates them to a specific environmental impact category. For example, if a gas is emitted, its emissions will contribute to categories such as climate change, air pollution or tropospheric ozone depletion among others, depending on its characteristics and the impact assessment weighting. Our primary outcome was climate impact, expressed as global warming potential in grams of carbon dioxide equivalents (CO_2_e) for a 100‐year time horizon. The secondary outcomes were the remaining environmental impacts.

### 2.8 Sensitivity Analyses

The sensitivity analyses evaluated the impact of the number of uses of the Aura40, the relative load in the cleaning and sterilisation process and a scenario without recycling. We also assessed the impact of using various European electricity mixes in the reprocessing of the Aura40. To enable comparisons with a previous study from the US, we performed an analysis using a US electricity mix [8]. The electricity data were obtained from the most recent data on electricity mixes that were available in ecoinvent for the respective countries (2020 for European nations and 2021 for the US). Because the newer version of our autoclave is more efficient, we also modelled a scenario with a more energy‐efficient autoclave—entailing 40% less electricity and water consumption—to reflect improvements in sterilisation technology.

### 2.9 Statistics

The ecoinvent database provides a distribution for the data points that reflect the uncertainties of the measurements. Wherever possible, the uncertainty of a data point is based on the variation in the sampled data. If a data point is derived from a single source without information on uncertainties, the ecoinvent database has a simplified procedure to estimate the uncertainty [14].

To assess overall uncertainty of the LCA model, we conducted Monte Carlo simulations (*n* = 1000) in which randomly selected input data within the uncertainty range for each parameter in the model, was used to simulate the median and 95% reference interval [15, 16]. As has been suggested for Monte Carlo simulations, inferential statistics were not used to compare the LMs [15]. Instead, dependent (paired) simulations were applied to assess the certainty of differences between products [15, 17]—i.e., the same sample of input data was used for shared processes in the various systems in each individual Monte Carlo run. Data were presented as medians with their 95% reference interval, unless stated otherwise. Differences between systems were considered when the 2.5th–97.5th percentile range of the differences did not include 0 [18].

## 3 Results

### 3.1 Life Cycle Inventory

Table 1 summarises the life cycle inventory; additional details are available in the Appendix.

**TABLE 1:** Summarised life cycle inventory of the laryngeal masks.

	Aura40	AuraStraight	Igel+
Size	5	5	4
Use (*n*)	40	1	1
Weight of mask (g)	71	63	89
Body material	Silicone	PVC	SEBS
Connector material	Polysulphone	PCTG	ABS
Packaging weight (g)	11 (one use)	22	39
Packaging material	Plastic/paper	Plastic	Plastic
Bulk packaging and manual (g)	579 (40 uses)	297 (10 masks)	342 (10 masks)
Packaging material	Board/paper	Board/paper	Board/paper
Production site	China	China	Lithuania
Transport (km)	19,569 (40 uses)	19,569	1601
Solid waste weight (g)	27	114	162
Soaking			
Load actual/max	10/10	NA	NA
Water (L)	10	NA	NA
Detergent (L)	0.020	NA	NA
Automated cleaning			
Load actual/max	7/10	NA	NA
Water (L)	19	NA	NA
Electricity (kWh)	4.2	NA	NA
Detergent (L)	0.058	NA	NA
Sterilisation			
Load actual/max	60/72	NA	NA
Water (L)	447	NA	NA
Electricity (kWh)	21.5	NA	NA
Cumulative consumption per laryngeal mask			
Detergent per use (L)	0.010	NA	NA
Water/wastewater per use (L)	11	NA	NA
Electricity per use (kWh)	0.944	NA	NA

*Note:* The values for Aura40 are for 1 of 40 uses unless stated otherwise.

Abbreviations: ABS, acrylonitrile butadiene styrene; NA, not applicable; PCTG, poly cyclohexylenedimethylene terephthalate glycol; PVC, polyvinylchloride; SEBS, styrene ethylene butylene styrene.

### 3.2 Climate Impact

**FIGURE 2:**
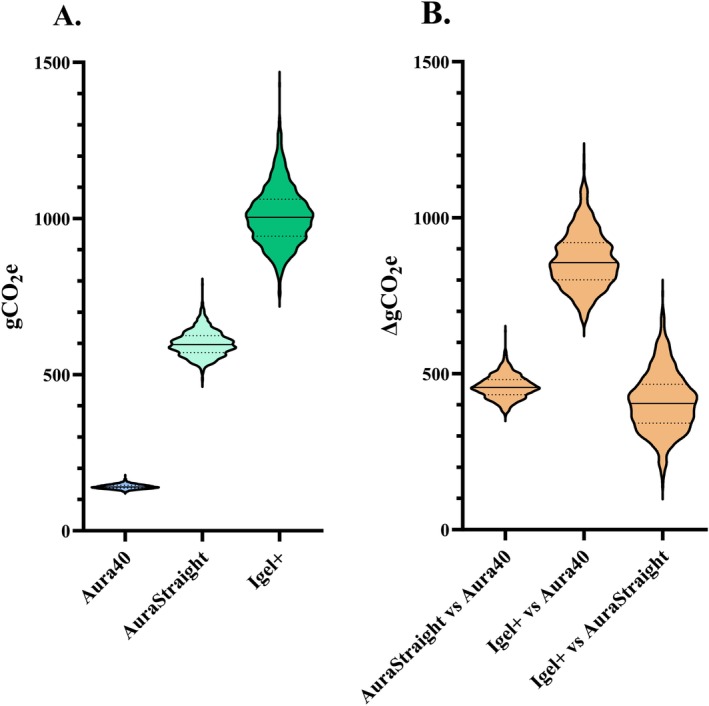
(A) Climate impact of laryngeal masks. (B) Difference in climate impact between laryngeal masks (gCO_2_e: gram carbon dioxide equivalent over a 100‐year period). The violin plot shows median, lower quartiles and upper quartile.

The climate impact of the Aura40 was 141 gCO_2_e (129–156), whereas that of the AuraStraight and Igel+ single‐use LMs was 597 gCO_2_e (533–686) and 1000 gCO_2_e (848–1210), respectively. The AuraStraight had a 323% greater impact on climate impact (median difference (∆) 456 gCO_2_e [390–535]) and the Igel+ had a 607% higher climate impact (∆856 gCO_2_e [709–1070]) versus the Aura40 (Figure 2). Compared with the AuraStraight, the Igel+ had a 68% higher impact on climate change (∆404 gCO_2_e [224–608]).

### 3.3 Other Environmental Impacts

Relative to the single‐use LMs, the Aura40 had a lower impact in several categories, including fine particulate matter, fossil resource scarcity, eutrophication, human carcinogenic toxicity, ozone formation, stratospheric ozone depletion, terrestrial acidification and toxicity. Conversely, the Aura40 had a higher impact on ionising radiation compared with the single‐use LMs. Of the single‐use LMs, the AuraStraight had less of an impact than the Igel+ on fossil resource scarcity and stratospheric ozone depletion. The Igel+ had a lower impact on ionising radiation than the AuraStraight. Complete results for the other environmental impact categories are presented in the Appendix.

### 3.4 Contribution Analysis

**FIGURE 3:**
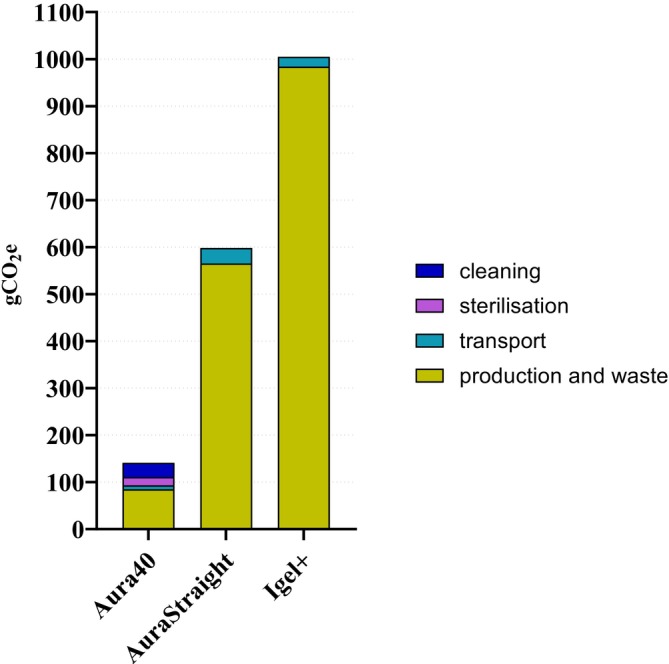
Contribution analysis of the climate impacts of laryngeal masks (gCO_2_e: gram carbon dioxide equivalent over a 100‐year period).

In the contribution analysis, the major climate impact of the Aura40 was attributed both to the production and destruction phases (51%) and the reprocessing phase (44%), whereas the production and destruction phases were the major contributors to that of the single‐use alternatives (95% and 98%, respectively) (Figure 3).

### 3.5 Sensitivity Analyses

The effects on the climate impact of the reusable and single‐use LMs with various electricity mixes are presented in Table 2. The Aura40 had a similar impact as the AuraStraight when modelled with the US electricity mix. With Czech and Polish electricity mixes, the Aura40 had a 19% and 66% higher impact versus the AuraStraight, respectively. The Igel+ had a similar impact as the Aura40 with the Polish electricity mix. With a Norwegian electricity mix, the Aura40 had a 9% lower climate impact than with the Swedish electricity mix that was used in the main model.

**TABLE 2:** Sensitivity analysis: influence of electricity mix on the climate impact of the Aura40 reusable laryngeal mask.

Laryngeal mask (electricity mix)	Climate impact of electricity (gCO_2_e/kWh)	Laryngeal mask climate impact (gCO_2_e)	Difference from Aura40 (gCO_2_e)	Difference from AuraStraight (gCO_2_e)	Difference from Igel+ (gCO_2_e)
Aura40 (Norway)	25	129 (117; 145)	12 (3; 20)	467 (401; 542)	868 (728; 1080)
Aura40 (Sweden)	38	141 (129; 156)	NA	456 (390; 535)	856 (709; 1070)
Aura40 (France)	90	189 (174; 208)	−48 (−62; −36)	405 (343; 488)	803 (660; 1010)
Aura40 (Spain)	212	305 (272; 343)	−163 (−205; −132)	292 (223; 374)	694 (543; 910)
Aura40 (EU)	334	421 (396; 452)	−279 (−303; −258)	177 (110; 255)	585 (423; 782)
Aura40 (Germany)	398	479 (438; 561)	−339 (−387; −297)	116 (36; 206)	519 (361; 710)
Aura40 (US)	486	562 (511; 625)	−421 (−484; −370)	33 (−52; 118)^a^	439 (280; 661)
Aura40 (Czechia)	641	709 (636; 792)	−571 (−637; −500)	−113 (−213; −13)	287 (124; 473)
Aura40 (Poland)	947	993 (872; 1150)	−854 (−1010; −737)	−395 (−555; −254)	8 (−200; 254)^a^
AuraStraight	NA	597 (533; 686)	−456 (−390; −535)	NA	404 (224; 608)
Igel+	NA	1000 (848; 1210)	−856 (−709; −1070)	404 (224; 608)	NA

*Note:* Electricity mixes are from 2022 for the US and from 2020 otherwise (Ecoinvent 3.10). Differences are expressed as median with 95% reference interval (2.5%; 97.5% percentiles); positive values for differences indicate a lower climate impact for the Aura40. Coloured background: main analysis results. gCO_2_e: gram carbon dioxide equivalent over a 100‐year period.

^a^ No difference; NA: not applicable.

The sensitivity analysis of the impacts of disinfector and autoclave loads and the number of reuses of the Aura40 is presented in the Appendix. If the load of the disinfector and autoclave is reduced below 10% the climate impact of the Aura40 is higher than for the AuraStraight. If the load of the disinfector and autoclave is reduced below 5% the climate impact of the Aura40 is higher than for the Igel+. When the disinfector and autoclave loads increased from 70% and 83%, respectively, to 100%, the climate impact of the Aura40 decreased by 8%. An autoclave with 40% lower electricity consumption reduced the climate impact of the Aura40 by 6% compared with the baseline scenario.

If reused at least 5 and 8 times, the Aura40 had a lower climate impact than the Igel+ and AuraStraight, respectively. Increasing the number of reuses to 80 reduced this climate impact by 31%. In the absence of recycling of the packaging, the climate impact of the Aura40 and AuraStraight increased by 8%; the impact of the Igel+ was unaffected by the assumption of recycling.

## 4 Discussion

In a Swedish setting, the reusable LM, Aura40, had a lower climate impact than the single‐use alternatives. However, the results were sensitive to the electricity mix, and in countries with a high‐climate‐impact electricity mix, the Aura40 had a greater climate impact than the single‐use LMs. Among the single‐use LMs, the Igel+ had a greater climate impact than the AuraStraight.

These findings align with earlier research from North America that has indicated the environmental benefits of reusable LMs [7]. However, this US study reported that the reusable LM had a lower climate impact despite the fossil‐heavy electricity mix of the US, which contrasts with our findings. This disparity in results is unexpected, given that the US study modelled an autoclave with higher electricity consumption and a lower load than in our study. Overall, the US study used 2.5 times more water and 4 times more electricity per LM during reprocessing than we did—factors that would be expected to increase the climate impact of reprocessing the reusable LM. Further, our model included the bulk packaging, which was heavier for the reusable mask than for the single‐use LM, compounding its climate impact.

We speculate that the discrepancy in findings is attributed to differences in the software and impact assessment methods that were used between studies [8]. The Building for Environmental and Economic Sustainability v4.02 impact assessment method was used in the previous study, whereas we used the ReCipe 2016 impact assessment method [19]. Both approaches are based on the International Panel on Climate Change climate impact method, which has undergone two updates since 2012. In addition, differences in allocation methods and our use of a more recent version of the ecoinvent database may also have contributed to the difference in results.

Our findings suggest that the climate impact of reusable LMs is highly dependent on the electricity mix. In certain contexts, particularly those with high‐carbon electricity, a single‐use LM, such as the AuraStraight, may be the more sustainable option with regard to climate impact. However, the climate impact of electricity is falling globally [20]. In 2024, the carbon impact of national electricity mixes was below 300 gCO_2_e/kWh in the UK and in 21 of the 27 EU countries, compared with 400 gCO_2_e/kWh in the US and 600 gCO_2_e/kWh in China [20]. This trend will increasingly favour the reusable option over the single‐use alternative. Our results indicate that 400 gCO_2_e/kWh and 700 gCO_2_e/kWh are the approximate thresholds for the climate impact of an electricity mix, below which the Aura40 has a lower climate impact than the AuraStraight and Igel+, respectively. Combined with our finding that the reusable LM has a similar or lower impact than the single‐use LMs in most other environmental categories, we conclude that it is the most environmentally friendly option in most European countries.

Our contribution analysis highlights several opportunities for further mitigating the environmental impacts of LMs. The main environmental impacts of single‐use LMs stem from the production and destruction phases, which involve fossil‐derived plastics, a fossil‐heavy electricity mix and plastic incineration—all of which contribute to air pollution and climate change. Thus, the environmental impacts of single‐use LMs could be reduced by decreasing the weight of material and packaging, using fossil‐free materials, applying decarbonised electricity mixes in production or increasing the recyclability of LMs.

To further decrease the climate impact of the reusable LM, previously published data and our sensitivity analysis suggest that efforts should aim to optimise washer‐disinfector and autoclave loading, increase the number of reuses and integrate more energy‐efficient equipment [21]. These strategies would lower its climate impact and other environmental impacts, such as water use, ionising radiation from nuclear power and land use from biomass exploitation.

In addition to their environmental impacts, the clinical efficacy and safety of LMs will influence clinical decisions. The choice of LM is commonly guided by perceived differences in the incidence of postoperative sore throat, oropharyngeal leaks and first‐attempt insertion failures. Two recent network meta‐analyses of randomised trials suggested that the Ambu AuraOnce LM performs similarly to the Igel with regard to these outcomes [22, 23]. Given that the Ambu AuraOnce LM is the single‐use version of the Aura40 and that the Igel and the Igel+ are anatomically very similar, we conclude that the Aura40 and Igel+ are likely to be clinically equivalent in common settings.

Regarding safety, observations of protein deposits that remain after reusable LMs are cleaned have raised concerns about the potential transmission of spongiform encephalopathy (i.e., Creutzfeldt–Jakob disease), leading to the common perception that single‐use LMs are safer than their reusable counterparts [4, 5, 24]. To date, approximately 400 cases of iatrogenic Creutzfeldt–Jakob disease have been reported, most of which were associated with organ transplants or treatment with hormone preparations from infected patients. There is no evidence to support that protein deposits on LMs can transfer the disease. Given that single‐use LMs contribute to environmental damage that affects human health through their environmental impacts and that the transmission of Creutzfeldt–Jakob disease via reusable LMs remains a theoretical risk, it is worth questioning whether such safety concerns over disease transmission merit the choice of a single‐use over a reusable LM.

At present, few, if any, reusable LMs are used in Sweden. Assuming that the national use of LMs is similar to that in our region, a full transition to reusable LMs could reduce the annual national climate impact of LMs in Sweden by approximately 80% (−70 tonCO_2_e) and lower LM‐related waste by 83% (16 t), as detailed in the Appendix. For perspective, a typical surgical procedure is reported to have a climate impact of 0.02–0.5 tonCO_2_e, and the total annual climate impact of halogenated anaesthetic gases that were administered in Sweden in 2023 was 1561 tonCO_2_e [25, 26].

## 5 Limitations

The data in the ecoinvent database are accompanied by associated uncertainty ranges, which are explored through Monte Carlo simulations. However, these uncertainty ranges are not always based on direct measurements—some are derived from estimates, and in certain cases, uncertainty data may be missing. Thus, the uncertainty in our results may not fully reflect reality and is likely to be underestimated. Further, some impact categories, such as water use, have high uncertainties, rendering comparisons between products less reliable. It could be argued that an increase in the number of simulations would have given us a more precise estimate of the uncertainty. However, increasing the number of simulations from 1000 to 10,000 only had a marginal effect (< 1%) on the 95% reference interval values at the expense of longer computing times [27].

Because manufacturers of LMs do not share details on their production processes, our analysis is based largely on assumptions and generic data from the ecoinvent database which may not reflect the materials and processes used in the actual factories. Moreover, some materials were not included in our analysis, such as colorants or plasticisers. However, because these represent a very low mass percentage of the total product, we believe that this omission most likely had a limited influence on our results. Consequently, our results may not reflect potential efforts by manufacturers of LMs to reduce their environmental impact, such as switching from fossil to renewable energy sources or using more efficient equipment. This limitation highlights the need for such producers to increase transparency by publicly disclosing their production routines or publishing an ISO 14025 ‘Environmental Product Declaration’, thereby enabling accurate and transparent comparisons between clinical products.

## 6 Conclusion

The Ambu40 reusable LM has a lower climate impact than the single‐use AuraStraight and Igel+ in settings with low‐climate‐impact electricity, such as Sweden. In contrast, the single‐use AuraStraight may have a lower climate impact than the Aura40 in countries with high‐climate‐impact electricity mixes. Among single‐use options, the Igel+ has a greater impact than the AuraStraight. The environmental impact of LMs should be considered in procurement decisions and may represent an opportunity to reduce the environmental burden of anaesthesia without compromising quality of care.

## Author Contributions

Conceptualisation: P.B. Data curation: A.T. Formal analysis: A.T., G.L., P.B. Funding acquisition: A.T., P.B. Investigation: A.T., G.L. Methodology: A.T., G.L., A.P., P.B. Resources: P.B. Supervision: P.B. Visualisation: A.T., P.B. Writing: A.T., G.L., P.B. Review and editing: A.T., G.L., A.P., P.B.

## Ethics Statement

The study does not involve human subjects, and no ethical approval is needed.

## Conflicts of Interest

The authors declare no conflicts of interest.

## Supporting information


**Data S1:** Supporting Information.

## Data Availability

The data that support the findings of this study are available from the corresponding author upon reasonable request.
