# Supplementary material for: Climate Impact of Laryngeal Masks: Climate and Other Environmental Impacts of Reusable and Single‐Use Laryngeal Masks in Sweden
Source: Acta Anaesthesiol Scand. 2025 Nov 4;70(1):e70144. doi: 10.1111/aas.70144 (PMC12586968; doi:10.1111/aas.70144)
Supplement: Supplementary file 1 — Data S1: Supporting Information. [file AAS-70-0-s001.pdf]

Appendix

Climate and other environmental impacts of reusable versus single-use laryngeal mask in Sweden

Adrien Talbot, Gang Liang, Andrius Plepys, Peter Bentzer

Model	Part	Material	Quantity	Unit	Use (n)	Data source	Quantity source	Allocated process	Allocated material	Recycling	Waste	Other
**Aura40**	Body	Silicone	66.7	g	40	AMBU	measured	Injection moulding	Silicone	-	Plastic incineration	Size 5
	Connector	Polysulphone	3.62	g	40	AMBU	measured	Injection moulding	Polysulphone	-	Plastic incineration	-
	Valve	PE, PP, nitrile, SS	0.7	g	40	AMBU	measured	Injection moulding	Polysulphone	-	Plastic incineration	-
	Original packaging	Tyvek	8.01	g	40	AMBU	measured	Extrusion and thermoforming	HDPE	Mixed plastic	Plastic incineration	80% recycling
		Paper, PET, PE	6.23	g	40	AMBU	measured	Extrusion plastic film	PET	Mixed plastic	Plastic incineration	80% recycling
	Bulk packaging	Cardboard	232.59	g	40	Helsingborg	measured	-	Corrugated board box	Core board	-	100% recycling
	Log card	Printed paper	5.88	g	40	Helsingborg	measured	-	Printed paper	Paper	-	100% recycling
	Manual	Printed paper	326.64	g	40	Helsingborg	measured	-	Printed paper	Paper	-	100% recycling
	Soaking	Tap water	10	L	10	Helsingborg	measured	-	Tap water	-	Wastewater	-
		Detergent	0,020	L	10	Helsingborg	measured	-	Detergent; see specific table	-	Wastewater	Suma Med Super LPH
	Automated cleaner	Softened water	19	L	10	Helsingborg	Getinge manual	-	Softened water	-	Wastewater	-
		Detergent	0.056	L	10	Diversey	measured	-	Detergent; see specific table	-	Wastewater	Suma Med Super LPH
		Rinsing agent	0,004	L	10	Diversey	measured	-	Rinsing agent; see specific table	-	Wastewater	Suma Med Rinse Plus
		Electricity	4.2	kWh	10	Getinge	Getinge manual	-	Low-voltage electricity, SE	-	-	-
	Repackaging	Paper	5.76	g	1	Safety sheet	measured	-	Printed paper	Paper	Paper incineration	80% recycling
		PP/PET	4.33	g	1	Safety sheet	measured	Extrusion plastic film	PP (78%)/PET (22%)	Mixed plastic	Plastic incineration	80% recycling
		Label	0.82	g	1	Helsingborg	measured	-	Printed paper	Paper	Paper incineration	80% recycling
		Electricity	0.001	kWh	1	Helsingborg	calculated	-	Low-voltage electricity, SE	-	-	-
	Autoclave	Deionised water	447	L	72	Helsingborg	Getinge manual	-	Deionised water	-	Wastewater	-
		Electricity	21.5	kWh	72	Helsingborg	Getinge manual	-	Low-voltage electricity, SE	-	-	-
	Transport	Sea freight	18642.59	km	1	AMBU	sea-distance.org	-	Container ship, sea	-	-	CN-DE
		Lorry	926.8	km	1	AMBU	Google Maps	-	Lorry >32 ton Euro6	-	-	DE-SE-Helsingborg

Table 1: AMBU®Aura40 inventory data. The allocated processes have cut-off allocations and are market processes. PE: polyethylene, HDPE: high-density polyethylene; PP: polypropylene; SS: stainless steel; PET: polyethylene terephthalate; CN: China; DE: Germany; SE: Sweden.

Model	Part	Material	Quantity	Unit	Use	Data source	Quantity source	Allocated process	Allocated material	Recycling	Waste	Other
**Suma Med Super LPH**	Detergent	Potassium carbonate	48	g	1L	Diversey	Safety sheet		Potassium carbonate	-	-	Detergent density 1.15
		Potassium hydroxide	1.1	g	1L	Diversey	Safety sheet		Potassium hydroxide	-	-	
		Deionised water	950.9	g	1L	Diversey	Safety sheet		Deionised water	-	-	
		HDPE	32	g	1L	Diversey	measured	Blow moulding	HDPE	Mixed plastic	-	DE-SE
		Road transport	1032	km	1L	Diversey	Google Maps		Lorry >32 ton Euro6	-	-	
**Suma Med Rinse plus**	Rinsing agent	Ethoxylated alcohol	200	g	1L	Diversey	Safety sheet		Ethoxylated alcohol ae 7 and 11	-	-	Agent density 1.08
		Sodium cumenesulphonate	28	g	1L	Diversey	Safety sheet		Sodium cumensulphonate	-	-	
		Water	772	g	1L	Diversey	Safety sheet		Deionised water	-	-	
		HDPE	32	g	1L	Diversey	measured	Blow moulding	HDPE	Mixed plastic	-	
		Road transport	1032	km	1L	Diversey	Google Maps		Lorry >32 ton Euro6	-	-	DE-SE

Table 2: Detergent and rinsing agent inventory. The allocated processes have cut-off allocations and are market processes. HDPE: high-density polyethylene; DE Germany; SE: Sweden.

Model	Part	Material	Quantity	Unit	Use (n)	Data source	Quantity source	Allocated process	Allocated material	Recycling	Waste	Other
**AuraStraight**	Body	PVC	58.79	g	1	AMBU	measured	Injection moulding	PVC suspension	-	PVC incineration	Size 5
	Connector	PCTG	3.1	g	1	AMBU	measured	Injection moulding	PET	-	Plastic incineration	
	Valve	PP, PC, Silicone	0.69	g	1	AMBU	measured	Injection moulding	PET	-	Plastic incineration	
	Mask protection	?	10.5	g	1	-	measured	Injection moulding	HDPE	-	Plastic incineration	
	Packaging	PET	11.49	g	1	AMBU	measured	Extrusion and thermoforming	PET	Mixed plastic	Plastic incineration	80% recycling
		Tyvek	3.63	g	1	AMBU	measured	Extrusion and thermoforming	HDPE	Mixed plastic	Plastic incineration	80% recycling
	Bulk packaging	Cardboard	259.72	g	10	AMBU	measured	-	Corrugated board box	Core board	-	
	Manual	Paper	37.01	g	10	AMBU	measured	-	Printed paper	Paper	-	
	Transport	Sea Freight	18642.59	km	1	AMBU	sea-distance.org	-	Container ship, sea	-	-	CN-DE
		Lorry	926.8	km	1	AMBU	Google Maps	-	Lorry >32 ton Euro6	-	-	DE-SE-Helsingborg

Table 3: AMBU®AuraStraight inventory. The allocated processes have cut-off allocations and are market processes. PVC: polyvinylchloride; PCTG: poly cyclohexylenedimethylene terephthalate glycol; PP: polypropylene; PC: polycarbonate; PET: polyethylene terephthalate; HDPE: high-density polyethylene; CN China; DE: Germany; SE: Sweden.

Model	Part	Material	Quantity	Unit	Use (n)	Data source	Quantity source	Allocated process	Allocated material	Recycling	Waste	Other
**Igel+**	Body	SEBS	79.59	g	1	Intersurgical	measured	Injection moulding	ABS	-	Plastic incineration	Size 4
	Connector	ABS	6.68	g	1	Intersurgical	measured	Injection moulding	ABS	-	Plastic incineration	-
	Cap	PP	2.41	g	1	Intersurgical	measured	Injection moulding	PP	-	PP incineration	-
	Mask protection	PP	12.08	g	1	Intersurgical	measured	Injection moulding	PP	-	PP incineration	-
	Packaging	Plastic	25.12	g	1	-	measured	Extrusion and thermoforming	PET	Mixed plastic	PE incineration	80% recycling
		Tyvek	1.81	g	1	Intersurgical	measured	Extrusion and thermoforming	HDPE	Mixed plastic	PE incineration	80% recycling
	Bulk Packaging	Cardboard	207.27	g	10	-	measured		Corrugated board box	Core board	-	100% recycling
	Manual	Paper	134.28	g	10	-	measured		Printed paper	Paper	-	100% recycling
	Transport	Container ship, sea	1162	km	1	Intersurgical	sea-distance.org		Container ship, sea	-	-	LT-SE
		Road	439	km	1	Intersurgical	Google Maps		Lorry >32 ton Euro6	-	-	LT and SE

Table 4: Intersurgical® Igel+ inventory data. The allocated processes have cut-off allocations and are market processes. SEBS: styrene ethylene butylene styrene; ABS: acrylonitrile butadiene styrene; PP: polypropylene; PAT: polyethylene terephthalate; HDPE: high-density polyethylene; PE: polyethylene; LT Lithuania; SE: Sweden.

	Unit	Aura40	AuraStraight	Igel+	AuraStraight vs Aura40	Igel+ vs Aura40	Igel+ vs AuraStraight
**Fine particulate matter formation**	mg PM2.5-eq	274 (232;345)	747 (645;902)	765 (638;922)	465 (358;588)	487 (358;639)	23 (-124;180)
**Fossil resource scarcity**	g oil-eq	37 (32;46)	183 (150;230)	321 (254;406)	143 (117;188)	285 (218;366)	136 (71;227)
**Freshwater ecotoxicity**	g 1,4-DCB-eq	34 (19;67)	22 (14;39)	26 (18;43)	-12 (-47;+8)	8 (-43;14)	4 (-6;23)
**Freshwater eutrophication**	mg P-eq	67 (44;124)	171 (103;351)	177 (108;347)	101 (49;236)	105 (52;248)	5 (-46;69)
**Human carcinogenic toxicity**	g 1,4-DCB-eq	40 (22;82)	85 (44;194)	79 (45;146)	44 (17;121)	36 (14;86)	-8 (-56;21)
**Human non-carcinogenic toxicity**	g 1,4-DCB-eq	424 (-1970;2790)	496 (-8660;9420)	603 (-7710;8890)	181 (-6330;7120)	110 (-5920;7340)	65 (-2030;1850)
**Ionising radiation**	Bq Co-60-eq	127 (31;1010)	16 (4;135)	26 (5;265)	-107 (-904;-28)	-101 (-801;-26)	10 (1-113)
**Land use**	$dm^2 \times$ yr annual cropland-eq	2.9 (2.6;8.3)	3.5 (1.4;6.2)	3.2 (1.3;5.4)	0.64 (-0.56; 2.2)	0.4 (-0.8;18)	-0.2 (-1.9;1.2)
**Marine ecotoxicity**	g 1,4-DCB-eq	44 (26;83)	32 (22;55)	38 (27;60)	-15 (-55;+13)	-7 (-49;23)	6 (-8;31)
**Marine eutrophication**	mg N-eq	11 (8;15)	26 (20;37)	30 (25;36)	15 (10;25)	19 (14;25)	4 (-7;11)
**Mineral resource scarcity**	mg Cu-eq	1470 (1040;2280)	1560 (1080;2740)	1380 (1040;1940)	88 (-853;+117)	-115 (-969;537)	-163 (-784;141)
**Ozone formation, Human health**	mg NOx-eq	435 (371;518)	1430 (1190;1760)	1410 (1160;1740)	988 (796;1270)	980 (742;1270)	-16 (-369;363)
**Ozone formation, Terrestrial ecosystems**	mg NOx-eq	451 (385;536)	1510 (1260;1870)	1530 (1250;1890)	1060 (854;1360)	1080 (819;1390)	15 (-371;418)
**Stratospheric ozone depletion**	mg CFC-11-eq	0.13 (0.09;0.27)	0.43 (0.31;0.69)	0.67 (0.38;1.3)	0.30 (0.14;0.55)	0.53 (0.25;1.17)	0.23 (0.05;0.76)
**Terrestrial acidification**	mg SO2-eq	643 (538;840)	1630 (1410;1940)	1900 (1550;2350)	983 (717;1260)	1240 (876;1680)	260 (-94;719)
**Terrestrial ecotoxicity**	g 1,4-DCB-eq	2280 (1380;4420)	3690 (2150;8360)	3300 (2020;7360)	1370 (400;3930)	996 (188;2570)	-363 (-1670;205)
**Water consumption**	m^3 water-eq	0.5 (-17.3;+12.1)	0.0 (-0.4;+0.4)	0.0 (-0.4;+0.3)	-0.9 (-11.6;14.3)	-0.9 (-11.7;16.7)	-0.0 (-0.3;0.0)

Table 5: Environmental outcome results.

The values are presented as median with 95% reference interval (2.5%;97.5%). Cells that are shaded light blue favour the Aura40, those in light green favour AuraStraight, those in a deeper green favour the Igel+ and light orange indicates no difference.

-eq: equivalent; PM2.5: articulate matter 2.5 µm; 1,4-DCB: 1,3 dichlorobenzene; P: phosphorus; Bq Co-60: becquerel cobalt-60; $dm^2 \times$ yr annual cropland: square decimetre year annual cropland; N: nitrogen; Cu: copper; NOx: nitrogen oxides; CFC-11: trichlorofluoromethane; SO2: sulphur dioxide; m^3 water: cubic metre water.

Reference: Huijbregts MAJ, Steinmann ZJN, Elshout PMF, et al. ReCiPe2016: a harmonised life cycle impact assessment method at midpoint and endpoint level. *The International Journal of Life Cycle Assessment* 2017; **22**(2): 138-47.

Laryngeal mask	Parameter changed	Laryngeal mask climate impact (gCO2e)	Difference from Aura40 (gCO2e)	Difference from AuraStraight (gCO2e)	Difference from Igel+ (gCO2e)
**Aura40**	Autoclave with 40% less electricity	133 (122;147)	8 (6;10)	465 (401;552)	864 (716;1070)
**Aura40**	Main analysis	141 (129;156)	NA	456 (390;535)	856 (709;1070)
**Aura40**	Load 5%	788 (703;905)	-647 (-754;-564)	-194 (-329;-82)	210 (35;424)
**Aura40**	Load 10%	443 (397;500)	-302 (-353;-261)	154 (75;239)	553 (390;770)
**Aura40**	Load 100%	130 (118;145)	12 (10;14)	469 (405;552)	871 (719;1070)
**Aura40**	Reused 4 times	936 (848;1060)	-799 (-897;-719)	-343 (-454;-238)	56 (-115;274)
**Aura40**	Reused 5 times	763 (688;858)	-623 (-697;-557)	-168 (-256;-69)	91 (81;436)
**Aura40**	Reused 7 times	559 (508;624)	-417 (-476;-375)	37 (-40;116)#	441 (292;651)
**Aura40**	Reused 8 times	497 (443;559)	-356 (-397;-319)	98 (29;183)	499 (345;701)
**Aura40**	Reused 80 times	96 (88;108)	44 (40;51)	501 (430;596)	903 (747;1110)
**Aura40**	No recycling	153 (140;170)	-11 (-16;-7)	443 (381;528)	845 (692;1050)
**AuraStraight**	No recycling	643 (569;735)	503 (439;580)	-47 (-73;-16)	352 (182;572)
**Igel+**	No recycling	1080 (911;1310)	924 (765;1150)	-471 (-692;-291)	-59 (-292;167)#
**AuraStraight**	Main analysis	597 (533;686)	456 (390;535)	NA	404 (224;608)
**Igel+**	Main analysis	1000 (848;1210)	856 (709;1070)	404 (224;608)	NA

Table 6: Sensitivity analysis assessing the impact of cleaning load, autoclave, number of reuses and recycling on the climate impact of laryngeal masks.

Differences are expressed as median with 95% reference interval (2.5%;97.5%); positive values for difference indicate a lower climate impact for the laryngeal mask in the first column. Coloured background: main analysis results. gCO_2e: gram carbon dioxide equivalent over a 100-year period. #no difference. NA: not applicable.

Table 7: Estimated climate impact of reusable laryngeal masks in Sweden- an extrapolation of laryngeal mask use in Region Skåne to Sweden.

	Inhabitants	Number of laryngeal masks	Number of Igel	Number of other laryngeal masks similar to AuraStraight	Laryngeal masks per capita	Climate impact single-use ($tonCO_2e$)	Climate impact 100% substitution to reusable ($tonCO_2e$)	Climate impact difference ($tonCO_2e$)	Climate impact reduction	Waste weight single-use (ton)	Waste weight reusable (ton)	Weight difference (ton)	Weight reduction
**Region Skåne**	1,340,000	15965	4675	11290	0.012	11.415	2.251	9.164	80%	2.584	0.434	2.150	83%
**Sweden**	10,230,000	121882	35690	86192	0.012	87.147	17.185	69.961	80%	19.730	3.311	16.418	83%

The calculated values are extrapolated values based on the population difference. Cells in light yellow represent calculated values. Values for laryngeal mask use in Skåne and the population are from 2019.
Climate impact and waste weight per use of Aura40 (141 gCO_2e; 27 g); AuraStraight (597 gCO_2e; 114 g); Igel+ (1000 gCO_2e; 162 g). CO_2e: carbon dioxide equivalent over a 100-year period.

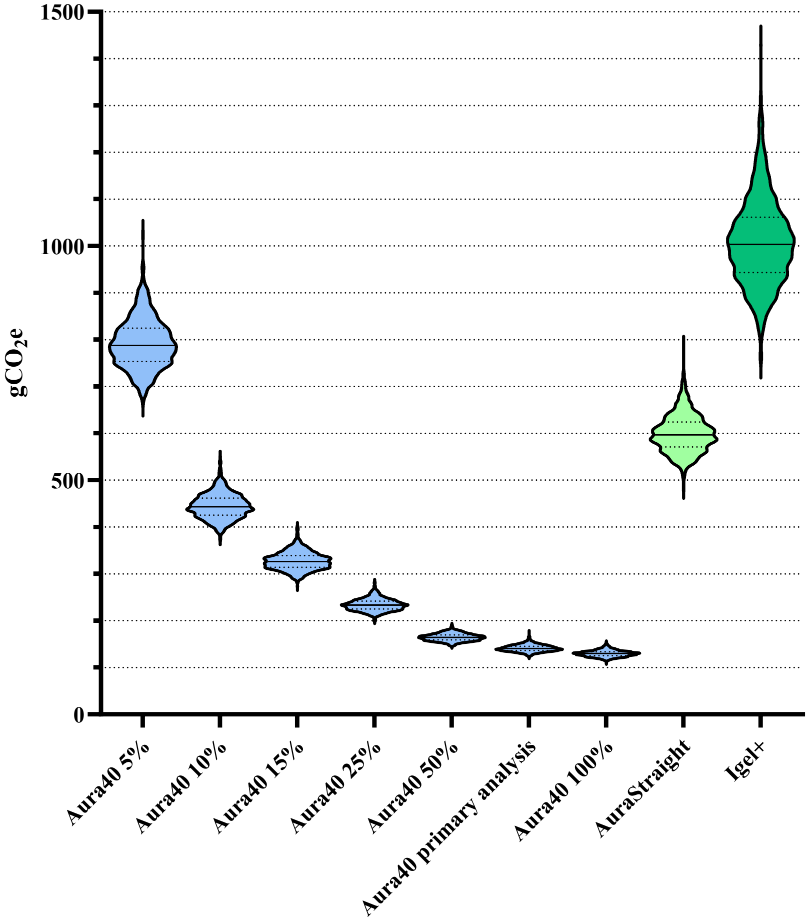

Figure 1: Sensitivity analysis assessing the impact of disinfector and autoclave load on the climate impact of the reusable laryngeal mask. In the primary analysis, the disinfector was loaded with 7 masks, with a maximum capacity of 10, and the autoclave was loaded with 60 masks, with a maximum capacity of 72 masks. Therefore, the Aura40 load was 70% in the disinfector and 83% in the autoclave in the primary analysis. The load sensitivity analysis shows the effect of load in the disinfector and autoclave in simulated scenarios, with similar loads in both machines ranging from 5% to 100%. gCO_2e: gram carbon dioxide equivalent over a 100-year period. The violin plot shows median, lower quartiles and upper quartile.

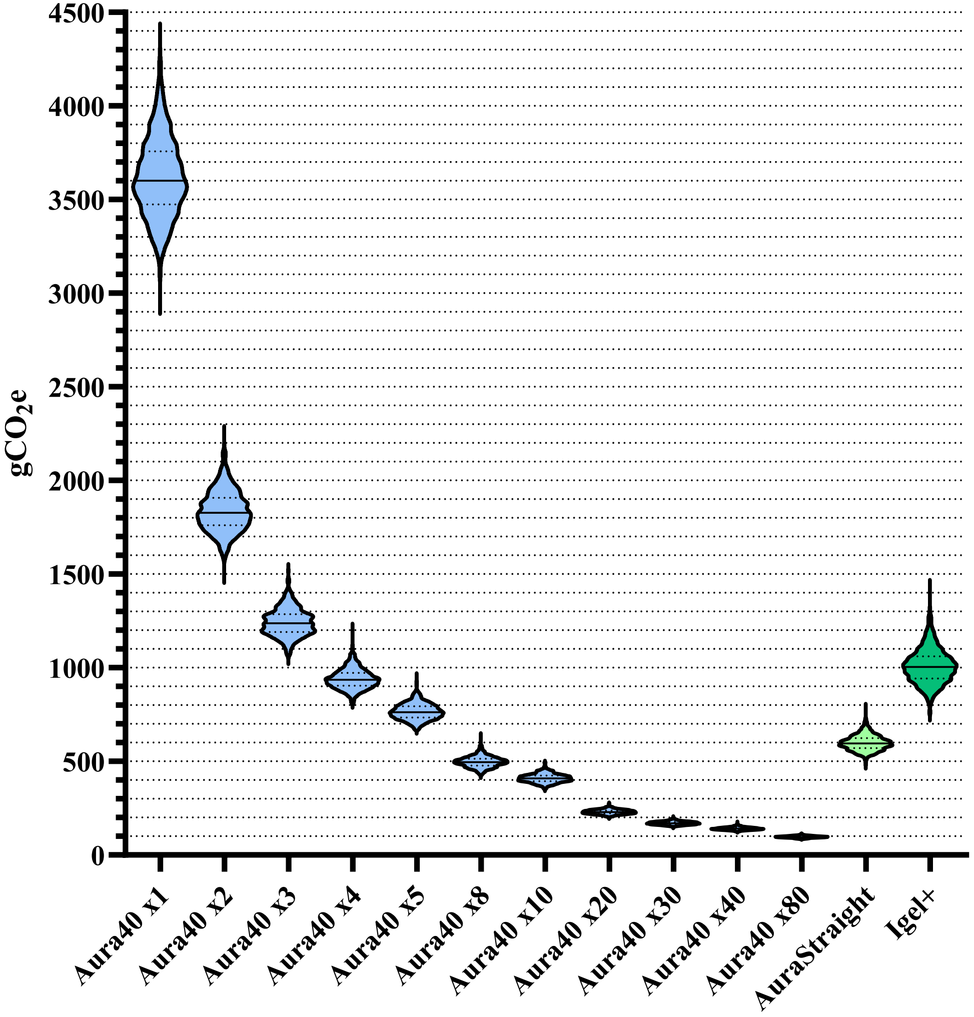

Figure 2: Sensitivity analysis assessing the impact of number of reuses on the climate impact of the reusable laryngeal mask. The primary analysis shows the maximum theoretical reuse of Aura40, which is 40 reuses. This sensitivity analysis presents the climate impact of the Aura40 with a reuse value of between 1 and 80 reuses before end of life. gCO_2e: gram carbon dioxide equivalent over a 100-year period. The violin plot shows median, lower quartiles and upper quartile.
